# Diverse Krill Lipid Fractions Differentially Reduce LPS-Induced Inflammatory Markers in RAW264.7 Macrophages In Vitro

**DOI:** 10.3390/foods10112887

**Published:** 2021-11-22

**Authors:** Dan Xie, Fangyuan He, Xiaosan Wang, Xingguo Wang, Qingzhe Jin, Jun Jin

**Affiliations:** 1 College of Biology and Food Engineering, Anhui Polytechnic University, Beijing Zhong Road, Wuhu 241000, China; xdwawj@163.com (D.X.); Hfy1124900348@163.com (F.H.); 2 Collaborative Innovation Center of Food Safety and Quality Control in Jiangsu Province, International Joint Research Laboratory for Lipid Nutrition and Safety, School of Food Science and Technology, Jiangnan University, 1800 Lihu Road, Wuxi 214122, China; wxstongxue@163.com (X.W.); wangxg1002@gmail.com (X.W.); jqzwuxi@163.com (Q.J.)

**Keywords:** krill oil, anti-inflammatory effect, RAW 264.7 cell, chemical composition

## Abstract

Antarctic krill oil is an emerging marine lipid and expected to be a potential functional food due to its diverse nutrients, such as eicosapentaenoic acid (EPA), docosahexaenoic acid (DHA), phospholipids, astaxanthin and tocopherols. Although krill oil has been previously proved to have anti-inflammatory activity, there is little information about the relationship between its chemical compositions and anti-inflammatory activity. In this study, the RAW264.7 macrophages model was used to elucidate and compare the anti-inflammatory potential of different krill lipid fractions: KLF-A, KLF-H and KLF-E, which have increasing phospholipids, EPA and DHA contents but decreasing astaxanthin and tocopherols levels. Results showed that all the krill lipid fractions alleviated the inflammatory reaction by inhibition of production of nitric oxide (NO), release of tumor necrosis factor-α (TNF-α), interleukin (IL)-1β and IL-6 and gene expression of proinflammatory mediators including TNF-α, IL-1β, IL-6, cyclooxygenase-2 (COX-2) and inducible nitric oxide synthase (iNOS). In addition, KLF-E with the highest phospholipids, EPA and DHA contents showed the strongest inhibition effect on the LPS-induced proinflammatory mediator release and their gene expressions. The results would be helpful to provide powerful insights into the underlying anti-inflammatory mechanism of krill lipid and guiding the production of krill oil products with tailor-made anti-inflammatory activity.

## 1. Introduction

Chronic inflammation is a common pathological process that is closely associated with the pathogenesis of various diseases, such as obesity, atherosclerosis, cachexia, asthma, metabolic disorders and diabetes [1,2,3]. Regulation of inflammation is particularly important for maintaining human health. Recently, there is an increasing concerning about the side effects and high cost of anti-inflammatory drugs that are clinically used [4]. Thus, the exploration of a natural food resource with potential anti-inflammatory activities has been gaining great interests in treatment of inflammation [5].

Antarctic krill oil is an emerging marine lipid and expected to be a potential functional food due to its diverse nutrients, such as eicosapentaenoic acid (EPA), docosahexaenoic acid (DHA), phospholipids, astaxanthin and tocopherols [6]. Krill oil manufacturers claimed that arthritic patients could benefit from supplementation of the lipid products [7]. Many studies have also investigated the anti-inflammatory effect of krill oil from the perspective of cell experiments [8,9,10], animal models [11,12] and human trials [7,13]. Results of these studies have preliminarily confirmed that krill oil showed anti-inflammatory activity, but there is no sufficient information on the contribution of different components to the anti-inflammatory activity. Some studies indicated the anti-inflammatory activity of krill oil was attributed to the EPA and DHA [11,14]. Indeed, dietary EPA and DHA have been extensively confirmed to effectively reduce proinflammatory responses by modulating nuclear factor-κB and regulating achidonic acid cyclooxygenase-derived eicosanoids, primarily prostaglandin E2-dependent signaling [15,16]. As is known, most EPA and DHA in krill lipids are bound to phospholipids [6], and marine phospholipids rich in n-3 PUFA were shown to have better bioavailability and anti-inflammatory properties than those of neutral n-3 PUFA [17]. However, in addition to EPA and DHA, astaxanthin and tocopherols are also known as physiologically active substances that have shown anti-inflammatory properties in some studies [18,19,20,21,22,23]. For example, Kimble et al. [20] found that pre-treatment with astaxanthin reduced transcriptional activation of nuclear factor-κB and activator protein-1, resulting in the downregulation of the production of inflammatory cytokines and mediators in SW-1353 human chondrosarcoma cells. An inhibition effect of phorbol myristate acetate-induced proinflammatory IL-1β expression was also observed when treated with tocopherols in human monocyte leukemic cell line THP-1 [21] as well as LPS-induced activation of rat Kupffer cells [22]. Unfortunately, most of current studies on the anti-inflammation of krill oil have ignored the contribution of these minor components to the overall anti-inflammatory effects of krill lipid products.

In addition, some studies have indicated that the extraction method could significantly affect the composition of krill oil, including the contents of phospholipids, EPA, DHA, astaxanthin and tocopherols [24,25,26]. This raised the question of whether krill oils with different lipid fractions would cause differences in their anti-inflammatory effects. In our previous study, a three-step extraction method was adopted to selectively extract three lipid fractions with different compositions, and they were confirmed to show significantly different antioxidant capacities [27]. Li et al. (2013) also pointed out that krill oil containing more phospholipids was more effective in decreasing plasma total cholesterol and low-density lipoprotein cholesterol levels in rats fed a high cholesterol diet [28]. However, there is a lack of literature focused on the anti-inflammatory activity discrepancy between krill oils with different lipid compositions.

Inflammatory responses induced by lipopolysaccharide (LPS) in macrophages have been commonly used as a classical model to evaluate the anti-inflammatory activity of active substances [29,30]. In this study, three krill lipid fractions with different compositions were prepared according to our previous study [27], and their anti-inflammatory properties were compared in LPS-activated RAW264.7 macrophages by analyzing the secretion of cytokines and gene expression associated with inflammatory responses. The results would be helpful to provide powerful insights into the underlying anti-inflammatory mechanism of krill lipid and guide the production of krill oil products with tailor-made anti-inflammatory activity.

## 2. Materials and Methods

### 2.1. Materials and Reagents

Krill meal was bought from Antarctic Farm Biotechnology Co., Ltd. (Jinan, China) and stored at −40 °C until used. Dulbecco’s modified Eagle’s medium (DMEM), fetal bovine serum (FBS), trypsin-EDTA solution and penicillin-streptomycin (P/S) were obtained from Gibco (Gaithersburg, MD, USA). Phosphate-buffered saline (PBS) was obtained from Hyclone (Logan, UT, USA). Lipopolysaccharide (LPS) (*Escherichia coli*, serotype 0111:04) was purchased from Solarbio (Beijing, China) and 3-(4, 5-dimethylthiazol-2-yl)-2,5-diphenyltetrazolium bromide (MTT) was bought from Sigma-Aldrich (St. Louis, MO, USA). The nitric oxide (NO) detection kit and R0026 RNAeasy kit were purchased from Beyotime (Nanjing, China). The enzyme immunoassay (EIA) kits for tumor necrosis factor (TNF)-α, interleukin (IL)-1β and IL-6 were obtained from MultiSciences (Hangzhou, China). TNF-α, IL-1β and IL-6, as well as inducible nitric oxide synthase (iNOS), cyclooxygenase-2 (COX-2) and GAPDH oligonucleotide primers were purchased from Sangon (Shanghai, China). The FastQuant cDNA kit was purchased from Qiagen (Gaithersburg, MD, USA). TaKaRa Premix Taq was purchased from TaKaRa (Otsu, Shiga, Japan), and iTaqTM SYBR^®^ Green SuperMix was obtained from Bio-Rad (Hercules, CA, USA). RAW 264.7 macrophages were purchased from American Type Culture Collection (Manassas, VA, USA). A Milli-Q apparatus (Billerica, MA, USA) was used to produce ultrapure water. Chromatographic grade solvents were bought from Sigma-Aldrich (St. Louis, MO, USA). All other analytical reagents were purchased from Sinopharm Medicine (Shanghai, China).

### 2.2. Preparation and Analysis of Three Krill Lipid Fractions with Different Compositions

Three fractions of krill lipid, namely KLF-A, KLF-H and KLF-E, were extracted using a three-step extraction method (sequentially using acetone, hexane and ethanol as the extraction solvent in each step) according to our previous study [27]. Briefly, in step 1, 100 g of krill meal was mixed with 200 mL of precooled acetone, and lipids were extracted at 4 °C for 15 min. The mixture was then filtered using a Buchner funnel. The KLF-A was recovered from the filtrate by removing acetone at 30 °C using a vacuum rotary evaporator. The residual krill meal (KM) was then dried and weighed as the extraction material for the next step (KM-H). In step 2, the lipid was extracted from the KM-H using hexane at 30 °C for 15 min. The ratio of KM-H to hexane was 1:2. KLF-H was obtained in similar manner to step 1, and the residual krill meal obtained in step 2 was also dried and weighed as the extraction material for the next step (KM-E). In step 3, we extracted KLF-E from KM-E using ethanol at 30 °C for 20 min at a 1:3 ratio (KM-E/ethanol, w:v). The amount of the lipid extracted in each step was recorded. The total lipid amount was also extracted from the same amount of krill meal by the Folch method [31] as a reference to calculate the lipid extraction efficiency in each step. The compositions of krill lipid fractions including the contents of phospholipids, astaxanthin and tocopherols, as well as the fatty acids profile, were analyzed referring to our previous studies [25,27]. Specifically, the determination of phospholipids’ content and composition was performed on a high-performance liquid chromatographic system (HPLC) (1260 Infinity, Agilent, Santa Clara, CA, USA) equipped with an evaporative light-scattering detector (ELSD). The amount of astaxanthin was determined by an HPLC (LC-20AT, Shimadzu, Kyoto, Japan) equipped with an ultraviolet detector (SPD-20A, Shimadzu, Kyoto, Japan) and a C18 column (5 μm, 4.6 × 250 mm; Hanbon, Huaian, Jiangsu, China) by comparing the peak area of the standard astaxanthin. The fatty acid composition was determined as fatty acid methyl esters (FAME) prepared according to our previous study [25] with a gas chromatographic system (GC) (7820A, Agilent, Santa Clara, CA, USA) equipped with a hydrogen flame ionization detector (FID) and a Trace TR-FAME capillary column (0.25 µm, 60 m × 0.25 mm; Thermo Fisher, Waltham, MA, USA), and the EPA and DHA contents were calculated as mg/g fraction by an external standard method.

The obtained krill lipid fraction was dissolved in DMSO with 2% tween 80 as reported by Kim et al. [32], and then the mixture was homogenized using an Ultra Turrax T25 blender (IKA, Staufen, Germany) at 12,000 rpm for 5 min. The mixture was used for cell experiments.

### 2.3. Cell Culture

RAW 264.7 was purchased from American Type Culture Collection (ATCC, Rockville, MD, USA). These cells were cultured in DMEM medium added with 10% heat-inactivated fetal bovine serum (FBS) and antibiotics composed of 100 U/mL of penicillin and 100 μg/mL of streptomycin and incubated in a 5% CO_2_ humidified environment at 37 °C (CO_2_ incubator, Heal Force).

The cells at 10–20 passages were normally cultured for 24 h, and then separately pretreated with the diverse krill lipid fraction at different concentrations. After 2 h, LPS (1 μg/mL) was directly added to stimulate inflammatory response for 24 h. The krill lipid fraction dissolved in DMSO with 2% tween 80 was diluted with DMEM to ensure the final concentration of DMSO with 2% tween 80 in culture medium less than 0.1%. In this study, the blank group refers to the cells cultured in the medium with no treatment, and the control group is those in the only LPS-stimulated medium. Both of the groups were treated with DMSO with 2% tween 80 of the same volume.

### 2.4. Cell Viability Assay

Cell viability treated with krill lipid fraction at different concentrations was measured by the methylthiazole tetrazolium (MTT) assay according to Mosmann et al. [33]. Briefly, after incubation with different concentrations (25–500 μg/mL) of krill lipid fraction and LPS stimulation (1 μg/mL) according to Section 2.3, 5 mg/mL of MTT working solution was added to the medium to dye the alive cells. After 4 h incubation, the supernatant was removed carefully, and DMSO was added into the well. Then, a Multiskan Go microplate reader (Thermo Scientific, Waltham, MA, USA) was used to determine the absorbance of each well at 490 nm. Cells treated with LPS alone were considered as the control group. The blank group was also set as the cells untreated, and its viability was taken as a reference. Cell viability was calculated as the following formula:

cell viability = (A_2_ − A_0_)/(A_1_ − A_0_) × 100% (1)

where A_0_ represents the absorbance of the group that did not contain cells, the sample and LPS; A_1_ represents the absorbance of the group that only contained untreated cells, without the sample or LPS; and A_2_ represents the absorbance of the sample group and control group.

### 2.5. Determination of NO Production

The NO content in the medium was measured using the Griess reaction by NO detection kit. After treatment with various concentrations of krill lipid fractions, RAW 264.7 cells were treated with or without LPS (1 μg/mL) for 24 h. The amounts of nitrite released in the cell cultures were determined as an indicator of NO release. Briefly, 100 μL of supernatant was reacted with 100 μL of Griess reagent, and the absorbance was determined at 540 nm using a Multiskan Go microplate reader (Thermo Scientific, Waltham, MA, USA). The NO production was calculated based on a standard sodium nitrite curve.

### 2.6. Cytokine Determinations

RAW 264.7 cells were cultured with different dosages of krill lipid fraction and then stimulated with LPS. Cytokine releases including IL-6, IL-1β and TNF-α in the culture medium were determined with EIA kits based on the manufacturer’s instructions.

### 2.7. Quantitative Real-Time Fluorescent PCR (RT-PCR) Analysis

#### 2.7.1. General

The levels of iNOS, COX-2, TNF-α, IL-6 and IL-1β mRNA expression were determined by RT-PCR.

#### 2.7.2. RNA Extraction and cDNA Synthesis

Total cellular RNA was extracted using R0026 RNAeasy kit (Beyotime, Nanjing, China) based on the manufacturer’s instructions. The RNA quality was evaluated by measuring the RNA quality index (RQI) on an Experion system (BioRad). All the samples reached an RQI higher than 7.0. The purity of extracted RNA was measured with a micro spectrophotometer (NanoDrop 2000, Thermo Scientific) and 260/280 indexes of all the samples were between 1.8 and 2.1. The quantity of total RNA was also determined with the micro spectrophotometer. Aliquots of isolated RNA (1 μg) from the samples were reverse transcribed into cDNA using the FastQuant cDNA kit (Gaithersburg, MD, USA) in a 25 μL reaction volume. One microliter of the obtained cDNA served as template for quantitative RT-PCR to quantify the relative mRNA content, and GAPDH was used as an internal control.

#### 2.7.3. Quantitative RT-PCR

Table 1 exhibited the oligonucleotide primers used in this study. Quantitative RT-PCR transcript levels were analyzed on a Bio-Rad CFX-Connect PCR instrument (Hercules, CA, USA) using iTaqTM Universal SYBR^®^ Green Supermix (Hercules, CA, USA). The relative mRNA expression levels of iNOS, COX-2, TNF-α, IL-6 and IL-1β transcripts were measured and calculated by the 2^−ΔΔCT^ method with GAPDH mRNA as the invariant control.

### 2.8. Statistics

All the experiments were conducted in triplicate in the same way. The data were displayed as mean ± standard deviation of replicated measurements. The significant differences (*p* < 0.05) were analyzed with SPSS software (version 19.0, SPSS, Inc., Chicago, IL, USA) by one-way analysis of variance (ANOVA) combined with Duncan’s multiple-range test (equal variances were assumed for the data) or the Games-Howell test (the heterogeneity for error variance was assumed for the data). All graphs were plotted with Origin 8.0.

## 3. Results and Discussion

### 3.1. Compositions of Three Krill Lipid Fractions

The three-step method adopted in this study resulted in different distributions of phospholipids and minor components in the three krill lipid fractions. As shown in Table 2, KLF-A extracted in the first step contained the lowest level of phospholipids but the highest concentration of astaxanthin and tocopherols. KLF-E was characterized by the highest phospholipids content and the least minor components. The levels of phospholipids and minor components of KLF-H fell between KLF-A and KLF-E. Additionally, the fractionation with high phospholipids content contained high EPA and DHA contents, as most of EPA and DHA are bonded to phospholipids [25]. The selective fractionation of krill lipids made it possible to illustrate the contributions of the diverse lipid components in krill to its anti-inflammatory activity.

### 3.2. Effect of Three Krill Lipid Fractions on the Viability of RAW264.7 Cells with LPS Stimulation

**Figure 1:**
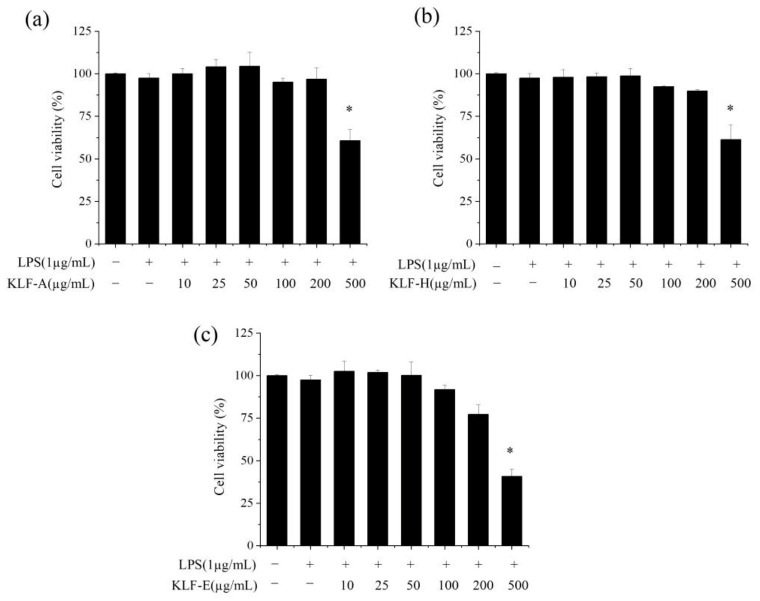
Effect of three krill lipid fractions with different concentrations on the cell viability of RAW264.7: (**a**) KLF-A, (**b**) KLF-H and (**c**) KLF-E. Blank group refers to the untreated cells and its viability was taken as reference (100%); cells treated with LPS alone were considered as the control group. * *p* < 0.05 versus the control group; one-way analysis of variance (ANOVA) combined with Games–Howell test was used to analyze the significance. Abbreviations: −, no addition; +, addition; LPS, lipopolysaccharide; KLF-A, the krill lipid fraction extracted with acetone in the first step; KLF-H, the krill lipid fraction extracted with hexane in the second step; KLF-E, the krill lipid fraction extracted with ethanol in the third step.

To evaluate and compare the potential anti-inflammatory effects of the three krill lipid fractions, RAW 264.7 macrophages stimulated with LPS were selected as the cell model. Firstly, appropriate dosage levels at which all the krill lipid fractions showed no cytotoxic need were determined to ensure enough cell viability. The effects of KLF-A, KLF-H and KLF-E at different concentrations in the culture (10, 25, 50, 100, 200 and 500 µg/mL) on the viability of RAW264.7 cells stimulated with LPS were investigated quantitatively, and the results are shown in Figure 1.

The results of MTT assay showed that RAW264.7 cell viabilities were reduced after LPS stimulation. Pre-incubation of all the three krill lipid fractions at 10~100 µg/mL before LPS stimulation could maintain better cell viability, but RAW264.7 cells showed the lowest tolerance to KLF-E. This may be attributed to the fact that the higher content of phospholipids in KLF-E increased the uptake of various components in krill lipid fractions by cells [34,35].

Based on the results of cell viability, the final concentrations administrated to RAW 264.7 cells of three types of krill oils were set at 25, 50 and 100 μg/mL in the medium for subsequent experiments.

### 3.3. Effect of Three Krill Lipid Fractions on NO Production in RAW264.7 Cells with LPS Stimulation

NO is one ubiquitous cellular molecule that is associated with many physiological and pathological processes [36]. Overproduction of NO may cause DNA damage, mitochondrial respiratory depression or react with superoxide anions to produce highly oxidized peroxynitrite anions, thereby affecting cell survival, forming inflammatory cascade waterfalls, promoting inflammation and leading to various diseases [37,38].

**Figure 2:**
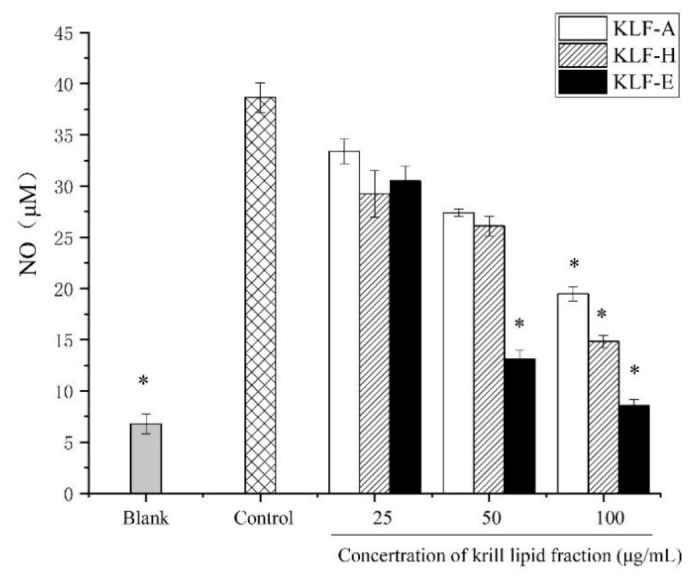
Effect of different concertrations of three krill lipid fractions on nitric oxide (NO) production in RAW264.7 cells with LPS stimulation. Blank group referred to the untreated cells; cells treated with LPS alone were considered as the control group. * *p* < 0.05 versus the control group; one-way analysis of variance (ANOVA) combined with Duncan’s multiple-range test was used to analyze the significance. Abbreviations: LPS, lipopolysaccharide; KLF-A, the krill lipid fraction extracted with acetone in the first step; KLF-H, the krill lipid fraction extracted with hexane in the second step; KLF-E, the krill lipid fraction extracted with ethanol in the third step.

The effects of KLF-A, KLF-H and KLF-E on NO production in LPS-stimulated RAW 264.7 cells are illustrated in Figure 2. As is shown, NO release in the control group significantly (*p* < 0.05) increased by up to six times in comparison to the blank group. Reductions of NO release induced by LPS were observed in different degrees when the cells were administrated to diverse krill lipid fractions at different dosages. For the specific treated group, the inhibition degree of NO production was positively correlated with the concentration of krill lipid fraction. However, different krill lipid fraction showed significant different in the inhibition of NO induced by LPS stimulation. It can be seen from Figure 2 that the NO production of LPS-induced inflammatory cells pretreated with 100 μg/mL of KLF-A, KLF-H and KLF-E decreased from 38.64 μM in the control group to 19.49, 14.83 and 8.56 μM, respectively. The NO production in the KLF-E group (8.56 μM) was almost the same as that in the blank group (6.78 μM) without LPS stimulation. Based on the results, KLF-E with the highest phospholipids content showed the strongest inhibitory effect on the NO production.

### 3.4. Effect of Three Krill Lipid Fractions on Cytokine Release and Related Gene Expression in RAW264.7 Cells with LPS Stimulation

**Figure 3:**
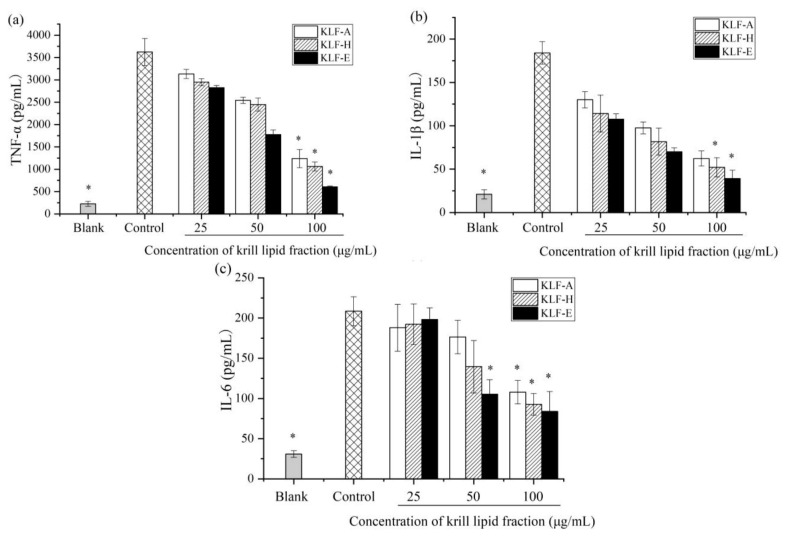
Effect of three krill lipid fractions on cytokine release in RAW264.7 cells with LPS stimulation: (**a**) TNF-α, (**b**) IL-1β and (**c**) IL-6. Blank group referred to the untreated cells; cells treated with LPS alone were considered as the control group. * *p* < 0.05 versus the control group; the one-way analysis of variance (ANOVA) combined with Games–Howell test was used to analyze the significance. Abbreviations: LPS, lipopolysaccharide; KLF-A, the krill lipid fraction extracted with acetone in the first step; KLF-H, the krill lipid fraction extracted with hexane in the second step; KLF-E, the krill lipid fraction extracted with ethanol in the third step.

When inflammation occurs, a series of cell pathways are activated and a variety of proinflammatory cytokines are releases. TNF-α, IL-1β and IL-6 are the most important proinflammatory cytokines produced by monocytes and macrophages, and their secretion levels can reflect the degree of inflammatory response [39,40,41,42,43,44]. In order to further explore the differences in the anti-inflammatory activities of the three krill lipid fractions, cytokine levels and gene expression including TNF-α, IL-6 and IL-1β were determined. The results are shown in Figure 3.

Figure 3a demonstrated the effect of three krill lipid fractions on TNF-α secretion in LPS-induced inflammatory cells. Stimulation with LPS in RAW 264.7 macrophages resulted in an order of magnitude higher TNF-α production (3623.89 pg/mL) compared with the blank group (226.11 pg/mL). It can be also seen that all of the groups treated with krill lipid fraction exhibited lower TNF-α production induced by LPS. For one specific treated group, a higher concentration of krill lipid fraction was used, and a higher reduction of TNF-α level was observed. At the highest dosage of 100 μg/mL, TNF-α secretion levels of the cells treated with KLF-A, KLF-H and KLF-E before LPS stimulation decreased to 1237.78, 1062.78 and 607.22 pg/mL, respectively. Bonaterra et al. has confirmed that krill oil attenuated the release of TNF-α by inhibiting the binding of LPS to toll-like receptor 4 using differentiated THP-1 macrophages activated by LPS [9]. A downregulation effect of TNF-α mRNA expression was also observed in inflammatory human colorectal adenocarcinoma cell lines (Caco2, HT29) [10]. Our results were consistent with these studies. Notably, the KLF-E group showed the most significant inhibitory effect on the TNF-α production, followed by the KLF-H and KLF-A group at the same concentration. Figure 3b,c shows the cytokine production of IL-1β and IL-6 in RAW 264.7 macrophages, respectively. Similar to TNF-α, levels of IL-1β and IL-6 in the control group were remarkably increased by 8.7- and 6.7-fold more than those in blank group. However, these levels were significantly decreased by krill lipid fraction treatment. Additionally, higher dosages of krill lipid fractions administrated to RAW 264.7 exhibited stronger inhibition effects on IL-1β and IL-6 production. There was a slight difference with the results of TNF-α that, at the lowest level of 25 μg/mL, the three krill lipid fractions provided insignificantly different IL-1β and IL-6 production levels, while KLF-E showed the strongest inhibition effect on IL-1β and IL-6 release among the three fractions at both 50 and 100 μg/mL.

**Figure 4:**
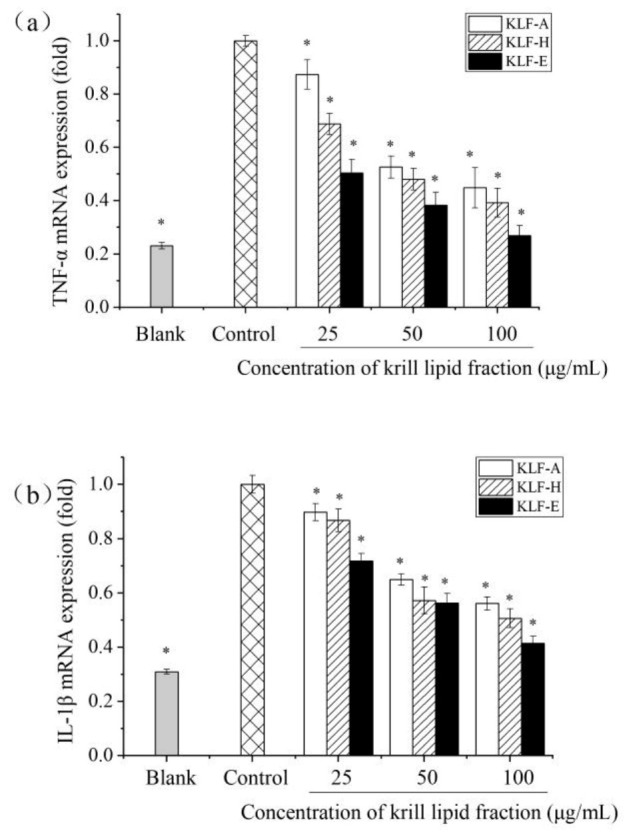

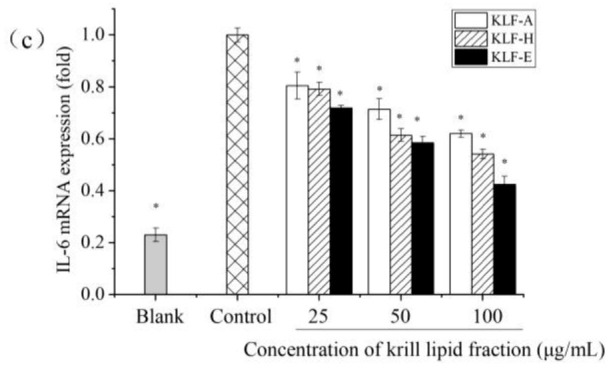
Effect of three krill lipid fractions on mRNA expression of cytokine (**a**) TNF-α, (**b**) IL-1β and (**c**) IL-6 in RAW264.7 cells with LPS stimulation. Blank group referred to the untreated cells; cells treated with LPS alone were considered as the control group. * *p* < 0.05 versus the control group; one-way analysis of variance (ANOVA) combined with Duncan’s multiple-range test was used to analyze the significance. Abbreviations: LPS, lipopolysaccharide; KLF-A, the krill lipid fraction extracted with acetone in the first step; KLF-H, the krill lipid fraction extracted with hexane in the second step; KLF-E, the krill lipid fraction extracted with ethanol in the third step.

Quantitative RT-PCR results of TNF-α, IL-1β and IL-6 mRNA expression are exhibited in Figure 4. Compared with blank group, the mRNA expression of TNF-α, IL-1β and IL-6 was significantly increased in LPS-stimulated cells, similar with their protein production in the cell culture shown in Figure 3. In contrast, cells preincubated with KLF-A, KLF-H and KLF-E had significantly decreased mRNA expression of the three proinflammatory cytokine upon stimulation relative to the control-stimulated group. Similar results were also found by Costanzo et al., who reported that the mRNA expression of IL-1β, IL-6 and IL-10 was decreased after administration with krill oil in C57BL/6 mice-induced colitis with dextran sodium sulphate (DSS) [45]. Ozonated krill oil also showed a reduction effect on the expression of the proinflammatory cytokines IL-1β and IL-6 at the mRNA expression level in LPS-induced RAW264.7 [32]. However, these studies did not provide the chemical compositions of the krill oil used. In this study, among the three krill lipid fraction-treated cells, KLF-E with the higher phospholipid, EPA and DHA contents showed significantly higher inhibition of mRNA expression of TNF-α, IL-1β and IL-6 compared to those of the other krill lipid fractions. These results were basically consistent with the inhibitory secretion of the three proinflammatory cytokines by the diverse krill lipid fractions.

### 3.5. Effect of Three Krill Lipid Fractions on Genes Expression of iNOS and COX-2 in RAW264.7 Cells

To further investigate the impact of three krill lipid fractions on proinflammatory cytokines, the mRNA expression levels of two important inducible enzymes in the inflammatory response, iNOS and COX-2, were also determined by q-PCR. The mRNA expression of both iNOS and COX-2 is limited under normal conditions. As previously mentioned, iNOS is highly expressed in the infected or inflammatory situation because a series of intracellular signaling pathways can be initiated and large amounts of NO are released at inflammatory sites. Additionally, as another important inducible enzyme, the transcription of COX-2 can be initiated in inflammatory response [46,47].

**Figure 5:**
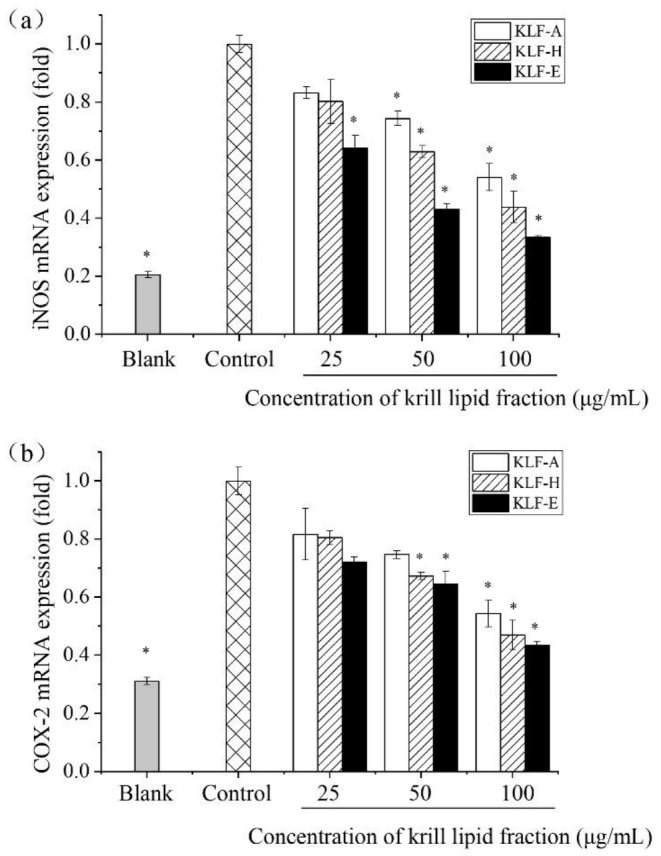
Effect of three krill lipid fractions on mRNA expression of inducible enzymes (**a**) iNOS and (**b**) COX-2 in RAW264.7 cells with LPS stimulation. Blank group referred to the untreated cells; cells treated with LPS alone were considered as the control group. * *p* < 0.05 versus the control group; one-way analysis of variance (ANOVA) combined with the Games–Howell test was used to analyze the significance. Abbreviations: LPS, lipopolysaccharide; KLF-A, the krill lipid fraction extracted with acetone in the first step; KLF-H, the krill lipid fraction extracted with hexane in the second step; KLF-E, the krill lipid fraction extracted with ethanol in the third step.

Figure 5 demonstrates the q-PCR results of iNOS and COX-2 mRNA expression. It can be seen that, compared with the blank group, the mRNA expression levels of iNOS and COX-2 were significantly increased after LPS stimulation (*p* < 0.05). After the intervention of krill lipid fraction, the gene expression of the two inducible enzymes decreased, and the downregulation effects were in a concentration-dependent manner for a certain krill lipid fraction. Additionally, a similar dose–response profile was observed for the three tested concentrations among the three lipid fractions. At the maximum administration concentration (100 μg/mL), the expression levels of iNOS in the cells treated with KLF-A, KLF-H and KLF-E were only 0.54, 0.44 and 0.33 times as much as those in the control group, respectively; the gene expression levels of COX-2 were 0.54, 0.47 and 0.43 times as much as those in the control group, respectively. These results indicated an effective downregulation effect of krill lipid fractions on the mRNA of iNOS and COX-2. Similar to other inflammatory markers, the higher the amount of phospholipids in the krill lipid, the more significant downregulation effect on the mRNA expression of iNOS and COX-2.

Unlike common edible oils that consist mainly of triacylglycerols, krill oil is characterized by a high phospholipids content and is rich in EPA and DHA. Moreover, some active lipid accompaniments, such as astaxanthin and tocopherol, are also present in krill [6], which further improves the nutrition value of krill oil. However, diverse processing methods have resulted in krill oil products with different compositions, and this limits our understanding of the association between composition and functionalities of krill oil. The present study was first undertaken to elucidate and compare the anti-inflammatory potential of different krill lipid fractions: KLF-A, KLF-H and KLF-E. These fractions mainly differed in the phospholipids content and minor components including tocopherols and astaxanthin, as well as the EPA and DHA contents. Indeed, n-3 PUFAs have demonstrated potent anti-inflammatory properties [15,16,48,49]. There has been some evidence showing that n-3 PUFAs exhibit anti-inflammatory properties by inhibiting both the IL-1, 2, 6 synthesis and the protein kinase C signaling pathway [50,51,52]. In our study, although to varying degrees, all the three krill lipid fractions containing n-3 PUFA-modulated proinflammatory cytokines IL-1β and IL-6 both at the protein expression and mRNA expression level in LPS-induced RAW264.7. Furthermore, some studies have reported that n-3 PUFA bound to phospholipids may be more bioactive and more bioavailable than n-3 PUFA as triacylglycerides [51,52]. It has been proven that EPA and DHA in krill lipids were mostly combined with phospholipids [6]. Thus, it is reasonable to hypothesize that lipid fractions with a high content of EPA and DHA as phospholipids would exert higher anti-inflammatory activity. The present study confirmed that the three krill lipid fractions with different compositions showed significant differences in the anti-inflammatory properties. Generally, KLF-E, which is the most abundant in EPA and DHA, was more favorable to reduce the production of NO, the secretion of inflammatory cytokines TNF-α, IL-1β and IL-6 and the expression of inflammatory-related genes in inflammatory cells. Especially at the highest concentration (100 μg/mL), KLF-E showed a more obvious superiority in anti-inflammatory responses compared to the other fraction lipids. The delivery efficiency of neutral oil into cell is limited because it is hard to dissolve in the culture and across membrane transport. Therefore, an oil-in-water emulsion has been considered as an effective delivery system of liposoluble components in cell experiments, and phospholipids were the commonly used emulsifiers for preparing emulsions [47,53]. In the present study, KLF-H and KLF-E contained a high content of phospholipids (35.02 and 62.79 g/100 g, respectively). In addition, EPA and DHA in krill lipids were mostly combined with phospholipids, which was beneficial to transport anti-inflammatory active factors EPA and DHA into cells. For KLF-A, although it contains high concentrations of the anti-inflammatory factors astaxanthin and tocopherol, it only contains 2.39 g/100 g of phospholipids. The low content of phospholipids indicates a low content of EPA and DHA, and this might also limit the transporting efficiency of other anti-inflammatory active components to cells, such as astaxanthin and tocopherol. In addition, there were slightly smaller differences between KLF-A, KLF-H and KLF-E in regulating NO production, inflammatory factor secretion and gene expression at the lowest administration concentration used in our study. Saw et al. found a synergistic anti-inflammatory effect of DHA/EPA with curcumin at low dosage in treating LPS-induced RAW264.7 cells [54]. In this study, although the contents of EPA and DHA in KLF-H were less than those in KLF-E, it contained a small amount of astaxanthin (30.03 mg/kg), while the astaxanthin content in KLF-E was very little (9.50 mg/kg). As a physiological active component with anti-inflammatory activity [55], the small quantity of astaxanthin and EPA/DHA in krill oil may also have a synergistic anti-inflammatory effect. However, when the dosage is increased to 50 and 100 μg/mL, this synergistic effect might be weakened by extremely high concentration of EPA/DHA. This could explain why KLF-E showed more significant anti-inflammatory activity than KLF-H and KLF-A at higher dosages (50 and 100 μg/mL), but less significant at 25 μg/mL. In addition, although there has been no direct evidence to support the synergistic anti-inflammation effect of n-3 PUFA and tocopherol, or tocopherol and astaxanthin, this is an interesting research topic to further illustrate the krill lipid anti-inflammation mechanism. Thus, more intensive study should be focused on the synergistic effect of different lipid components to support our results in future.

## 4. Conclusions

In this study, the LPS-stimulated RAW 264.7 macrophages model was used to evaluate the anti-inflammatory activities of the three krill lipid fractions with different compositions. The results clearly indicate that treatment of all the three krill lipid fractions decreased the production of LPS-induced proinflammatory mediators NO, TNF-α, IL-1β and IL-6, as well as the gene expression of proinflammatory cytokines and two inducible enzymes, iNOS and COX-2, in inflammatory cells. However, diverse krill lipid fractions differentially reduced LPS-induced inflammatory markers in RAW264.7 macrophages. Generally, KLF-E, with the highest phospholipids, EPA and DHA contents, showed the highest anti-inflammatory effect among the three krill lipid fractions. As most of EPA and DHA are associated with phospholipids, the results indicated that krill oil manufacturers should optimize technology to increase the phospholipids content if they want to produce products with tailor-made anti-inflammatory activity. However, the synergistic effect of n-3 PUFA and astaxanthin in anti-inflammatory activity still needs to be explored in future.

## Figures and Tables

**Table 1:** Primer sequences of the tested genes.

Gene	Forward (5′-3′)	Reverse (5′-3′)
GAPDH	ATG TAC GTA GCC ATC CAG GC	AGG AAG GAA GGC TGG AAG AG
IL-1β	CTG TCC TGC GTG TTG AAA	TTC TGC TTG AGA GGT GCT GA
IL-6	AGG AGA CTT GCC TGG TGA AA	CAG GGG TGG TTA TTG CAT CT
TNF-α	AGG CCT TGT GTT GTG TTT CCA	TGG GGG ACA GCT TCC TTC TT
iNOS	CAC CTT GGA GTT CAC CCA GT	ACC ACT CGT ACT TGG GAT GC
COX-2	TGA AAC CCA CTC CAA ACA CA	GAG AAG GCT TCC CAG CTT TT

**Table 2:** Compositions of the three krill lipid fractions used in this study ^1^.

Analytical Determination	KLF-A	KLF-H	KLF-E
Lipid yield (% d.b.)	5.23 ± 0.25 ^b^	5.01 ± 0.36 ^b^	8.97 ± 0.40 ^a^
Lipid extraction efficiency (% total lipid)	25.37 ± 1.21 ^b^	24.21 ± 1.72 ^b^	45.94 ± 2.03 ^a^
Phospholipids (g/100 g)	2.39 ± 0.11 ^c^	35.02 ± 2.06 ^b^	62.79 ± 2.45 ^a^
Astaxanthin (mg/kg)	519.80 ± 23.56 ^a^	30.03 ± 0.68 ^b^	9.50 ± 0.06 ^b^
Tocopherols (mg/100 g)	29.65 ± 0.52 ^a^	11.57 ± 0.45 ^b^	3.73 ± 0.35 ^c^
EPA (mg/g)	74.24 ± 4.31 ^c^	132.57 ± 8.97 ^b^	224.01 ± 9.97 ^a^
DHA(mg/g)	25.51 ± 3.17 ^c^	94.79 ± 7.24 ^b^	134.04 ± 7.34 ^a^

^1^ Abbreviations are: KLF-A, the krill lipid fraction extracted with acetone in the first step; KLF-H, the krill lipid fraction extracted with hexane in the second step; KLF-E, the krill lipid fraction extracted with ethanol in the third step; EPA, eicosapentaenoic acid; DHA, docosahexaenoic acid. Values are means ± standard deviation. d.b., dry basis of initial krill meal. The significant differences (*p* < 0.05) were analyzed with SPSS software (version 19.0, SPSS) by one-way analysis of variance (ANOVA). Different superscript letters in a row indicate significant differences (*p* < 0.05).
